# Comparison of virtual and in-person simulations for sepsis and trauma resuscitation training in Singapore: a randomized controlled trial

**DOI:** 10.3352/jeehp.2024.21.33

**Published:** 2024-11-18

**Authors:** Matthew Jian Wen Low, Gene Wai Han Chan, Zisheng Li, Yiwen Koh, Chi Loong Jen, Zi Yao Lee, Lenard Tai Win Cheng

**Affiliations:** 1 Emergency Medicine Department, National University Hospital, Singapore; 2 Department of Emergency Medicine, Ng Teng Fong General Hospital, Singapore; 3 Department of Emergency Medicine, Woodlands Health, Singapore; Hallym University, Korea

**Keywords:** Hospital emergency service, Patient simulation, Personal satisfaction, Sepsis, Singapore

## Abstract

**Purpose:**

This study aimed to compare cognitive, non-cognitive, and overall learning outcomes for sepsis and trauma resuscitation skills in novices with virtual patient simulation (VPS) versus in-person simulation (IPS).

**Methods:**

A randomized controlled trial was conducted on junior doctors in 1 emergency department from January to December 2022, comparing 70 minutes of VPS (n=19) versus IPS (n=21) in sepsis and trauma resuscitation. Using the nominal group technique, we created skills assessment checklists and determined Bloom’s taxonomy domains for each checklist item. Two blinded raters observed participants leading 1 sepsis and 1 trauma resuscitation simulation. Satisfaction was measured using the Student Satisfaction with Learning Scale (SSLS). The SSLS and checklist scores were analyzed using the Wilcoxon rank sum test and 2-tailed t-test respectively.

**Results:**

For sepsis, there was no significant difference between VPS and IPS in overall scores (2.0; 95% confidence interval [CI], -1.4 to 5.4; Cohen’s d=0.38), as well as in items that were cognitive (1.1; 95% CI, -1.5 to 3.7) and not only cognitive (0.9; 95% CI, -0.4 to 2.2). Likewise, for trauma, there was no significant difference in overall scores (-0.9; 95% CI, -4.1 to 2.3; Cohen’s d=0.19), as well as in items that were cognitive (-0.3; 95% CI, -2.8 to 2.1) and not only cognitive (-0.6; 95% CI, -2.4 to 1.3). The median SSLS scores were lower with VPS than with IPS (-3.0; 95% CI, -1.0 to -5.0).

**Conclusion:**

For novices, there were no major differences in overall and non-cognitive learning outcomes for sepsis and trauma resuscitation between VPS and IPS. Learners were more satisfied with IPS than with VPS (clinicaltrials.gov identifier: NCT05201950).

## Graphical abstract

[Fig f6-jeehp-21-33]

## Introduction

### Background/rationale

Technology-enhanced simulation encompasses multiple modalities, including in-person simulation (IPS) using mannequins, cadavers and animal tissue, virtual reality, and virtual patient simulation (VPS), which is “a specific type of computer-based program that simulates real-life clinical scenarios; learners emulate the roles of health care providers to obtain a history, conduct a physical exam, and make diagnostic and therapeutic decisions” [1]. Educators facing multiple options require evidence to inform instructional design choices to achieve the desired outcomes efficiently.

VPS presents several theoretical advantages over IPS. Cognitive load theory would predict that VPS is advantageous for novices learning complex resuscitations with high intrinsic load [2]. VPS would reduce the extrinsic load and titrate the intrinsic load to manageable levels. A single-player VPS would give every learner more opportunity for deliberate practice as the primary clinician for each resuscitation.

Although existing literature has compared VPS to IPS in areas such as recognizing clinical deterioration [3], there is little literature about VPS for sepsis or trauma resuscitation [4] which require integration of skills across multiple learning outcome taxonomy domains: cognitive, and non-cognitive, such as psychomotor and affective [5]. The existing VPS literature has focused on cognitive tasks of limited scope, with debatable generalizability to more complex and integrative tasks. The abovementioned benefits may be outweighed if functional task alignment is too far removed in VPS [6], where practice opportunities are limited for psychomotor skills and skills integration. The net result of these opposing effects is uncertain; nonetheless, this issue is important for informing instructional choices.

### Objectives

We sought to test the following hypotheses in novice learners of sepsis and trauma resuscitation: first, VPS would lead to overall better subsequent performance in simulated in-person resuscitation than IPS; second, differences between VPS and IPS would be driven by better cognitive learning with VPS, to the detriment of non-cognitive domains.

## Methods

### Ethics statement

Institutional review board approval was obtained from Singapore’s National Healthcare Group Domain Specific Review Board (DRSB reference number: 2021/00542). This study was registered with clinicaltrials.gov (trial identifier number: NCT05201950). Informed consent was obtained from 40 participants.

### Study design

This was a parallel-group randomized controlled trial comparing VPS and IPS. The study was described according to the CONSORT (Consolidated Standards of Reporting Trials) statement.

### Setting

This study was conducted from January to December 2022 at the Emergency Medicine Department, National University Hospital, Singapore.

### Interventions

Participants attended a lecture on resuscitation at the start of their emergency department rotation, followed by VPS or IPS. In IPS, 2–3 junior doctors worked with nurses in an in-situ simulation with the HAL (Gaumard Scientific) mannequin. Each session lasted 70 minutes and covered 1 sepsis resuscitation scenario and 1 trauma resuscitation scenario. To reduce bias, these were facilitated and debriefed by emergency department attending physicians who were not study team members, unaware of the study purpose, and incidentally scheduled for clinical duty during IPS days, per local norms.

A VPS session was scheduled close to each IPS date to minimize confounding from time between intervention and assessment. Participants were instructed to complete 1 sepsis resuscitation scenario and 1 trauma resuscitation scenario on a virtual patient software (Full Code, Full Code Medical) using their personal computer or mobile phone as many times as they wished to attain the highest possible score within 70 minutes. The fifth author (C.L.J.) proctored participants over video conferencing, only ensuring that the time limit was not exceeded, with no other input to reduce bias. Case content, such as patient history and image cues, was identical to the IPS cases.

The VPS used in this study was a single-user simulation with an exploratory design and high autonomy. There were over 100 constantly available actions, such as selecting investigations, ordering treatment, and obtaining a history or physical examination for specific organ systems. Images and audio cues, such as cardiac rhythm monitor audio or X-ray images, were provided when ordered by participants. Participants were required to select the diagnosis and disposition. After each attempt, participants received automated scoring and feedback listing actions that were correct, missed, or unnecessary, and explanations for each. Time stamps of VPS activity were automatically recorded by the software.

### Participants

The participants were junior doctors in the emergency department at National University Hospital in Singapore, with a rotation duration of at least 4 months. Recruitment, interventions, and learning outcome assessment all occurred between January and December 2022. We excluded pregnant doctors for their safety, and those whose rotation schedules precluded feasible scheduling of the study procedures.

### Outcomes

The primary outcome was participants’ scores obtained in the outcome assessment simulation at the end of the 3rd month of junior clinicians’ emergency medicine rotation. The secondary outcome was learner satisfaction within 1 week after the completion of the assigned intervention.

### Data sources/measurement

Learning outcomes were assessed via 2 simulated resuscitation scenarios in a simulation laboratory with a high-fidelity mannequin (Sim-Man 3G, Laerdal Medical) at the end of each participant’s emergency department rotation. The 2 assessment scenarios were different from the intervention scenarios, but still covered resuscitation in trauma (hemorrhagic shock from pelvic fracture and splenic rupture) and sepsis (severe bacterial pneumonia with septic shock).

We used the nominal group technique (NGT) consensus process to generate marking checklists [7]. All study team members (n=7) participated in the NGT process, comprising 5 attending emergency physicians and 2 fourth-year residents in emergency medicine. The 10th edition Advanced Trauma Life Support Manual [8] and 2021 Surviving Sepsis Campaign Guidelines [9] were provided as references. Each member took turns to suggest checklist items until no further items were suggested. Items were refined through discussion. Members independently and anonymously rated each item as “remove,” “undecided,” or “keep.” Consensus was defined a priori as 70% agreement, and it was achieved for all items after 1 round of voting. Then, definitions of cognitive, psychomotor, and affective domains from Bloom [5] and Harrow [10] were given to members as references. For each checklist item, members voted for the domains involved. The aggregate data were fed back to members between rounds of voting and discussed. All items achieved consensus after 2 rounds. Checklist items are provided as supplementary material (Supplement 1).

Each assessment scenario was piloted with a team member (L.T.W.C.) portraying poor performance once and excellent performance once, to calibrate 2 raters (G.W.H.C. and M.J.W.L.), standardize simulated assistant actions, and determine required time. One simulated doctor (Z.L. or Z.Y.L.) and 1 simulated nurse (Y.K.) provided standardized assistance such as oxygen device application or blood taking only when ordered by the participant.

The participants had 14 minutes to complete each scenario. The raters directly observed in real time and independently marked each checklist item as “not done,” “done but inadequate or not timely,” and “done adequately and timely,” scoring 0 points, half a point, and 1 point, respectively.

Self-reported satisfaction was measured after IPS or VPS using the Student Satisfaction with Learning Scale (SSLS). This 5-item questionnaire has been externally validated in health professions education [11]. Demographic characteristics of each participant were collected with the SSLS. Research data were available from Dataset 1.

### Bias

There was no selection bias because all feasible subjects were included.

### Study size

This is a pilot study with no prior data to guide sample size calculation. Post hoc calculation using G*Power software ver. 3.1.9.7 (Heinrich-Heine-Universität Düsseldorf) showed a power of 0.22 and 0.09 with an alpha of 0.05 for sepsis and trauma assessment scenarios, respectively.

### Randomization

**Fig. 1.**
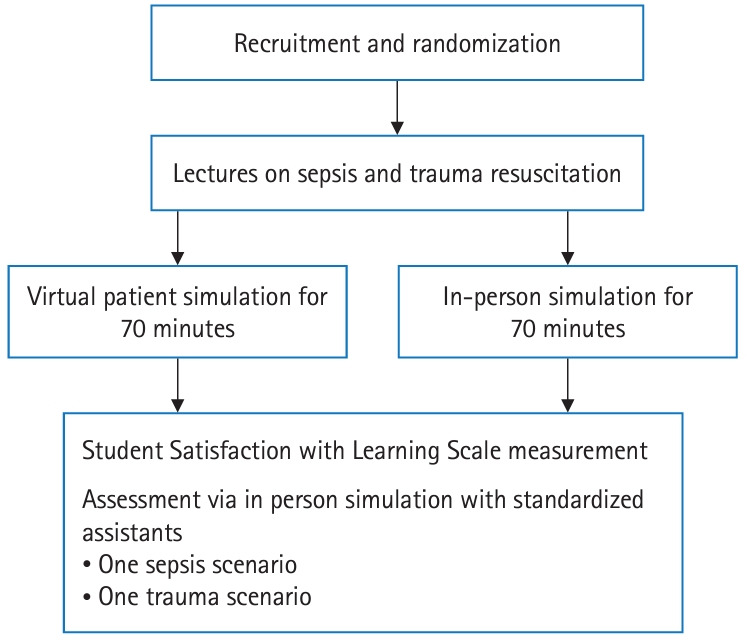
Sequence of interventions and assessments to determine the effect of virtual patient simulation versus in-person simulation on junior doctors learning sepsis and trauma resuscitation.

A randomized sequence allocating participants 1:1 to VPS or IPS groups was generated on STATA (Stata Corp.) by 1 researcher (L.T.W.C.) and placed in opaque sealed envelopes which were handed to another researcher (C.L.J.) who obtained informed consent from participants, then randomized them by opening envelopes according to recruitment sequence, and scheduled them for interventions (Fig. 1).

### Blinding

All researchers were blinded to participants’ group allocation, except the fifth author (C.L.J.), who did not participate in assessment scenarios and data analysis.

### Statistical methods

Means and standard distributions (SDs) are reported for normally distributed data, and medians and interquartile ranges (IQR) for non-normally distributed data. Reliability of the SSLS was measured using Cronbach’s α, and assessment checklist interrater reliability was measured using the intraclass correlation coefficient (ICC). Median SSLS scores were analyzed using the Wilcoxon signed rank sum test. Checklist items were grouped by consensus-determined domains. As all items were deemed to involve the cognitive domain in deciding to perform the action, items were grouped into those that were only cognitive and those that were not only cognitive. Checklist score means for each domain and summed across domains were analyzed with the 2-tailed t-test. Cohen’s d was calculated and interpreted as small if 0.2–0.5, moderate if 0.5–0.8, and large if >0.8 [12]. Data analysis was carried out by the seventh author (L.T.W.C.), blinded to group allocation, on BlueSky Statistics ver. 7.10 (Blue Sky Statistics LLC).

## Results

### Participant flow

**Fig. 2.**
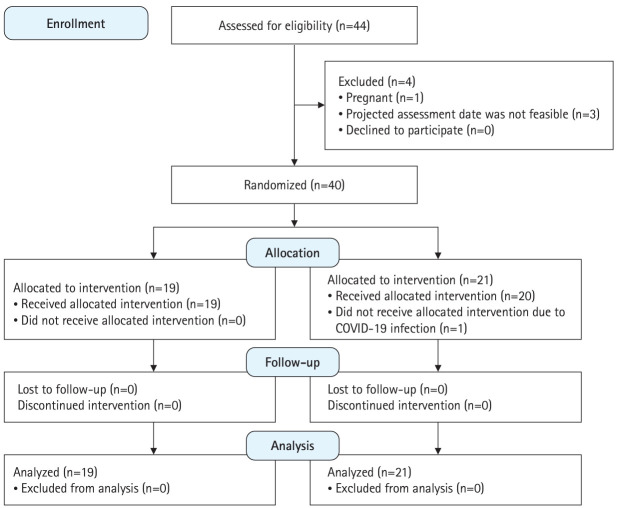
CONSORT (Consolidated Standards of Reporting Trials) flow diagram showing flow of participants through the study. COVID-19, coronavirus disease 2019.

All 44 junior doctors were screened from January to December 2022. One was excluded due to pregnancy. Three were excluded as their posting commencement date resulted in an unfeasible projected assessment date. Nineteen were randomized to VPS and 21 were randomized to IPS (Fig. 2). One participant did not undergo IPS due to coronavirus disease 2019 infection, and could not be rescheduled in time before undergoing assessment scenarios. This participant was retained in IPS for an intent-to-treat analysis.

### Main results

Baseline characteristics are provided in Table 1. Despite randomization and attempts to match scheduling for VPS and IPS, both groups were imbalanced. The VPS group had more men, with fewer years post-graduate, fewer months of prior emergency department experience, and a shorter median duration between intervention and assessment than the IPS group.

The median number of attempts at each VPS scenario was 2 (IQR, 1 to 3) for sepsis and 2 (IQR, 1 to 3) for trauma. The median total time spent in VPS was 45 minutes and 30 seconds (IQR, 35 minutes and 34 seconds to 48 minutes 22 seconds). Cronbach’s α for SSLS was 0.88, indicating good reliability. The ICC was 0.839 for checklists, indicating good interrater agreement.

**Fig. 3.**
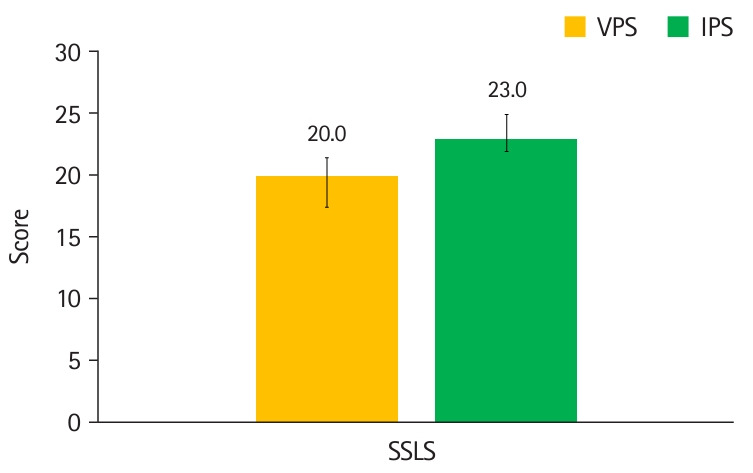
Comparison of Student Satisfaction with Learning Scale (SSLS) scores between virtual patient simulation (VPS) and in-person simulation (IPS) groups. Whiskers show interquartile ranges.

**Fig. 4.**
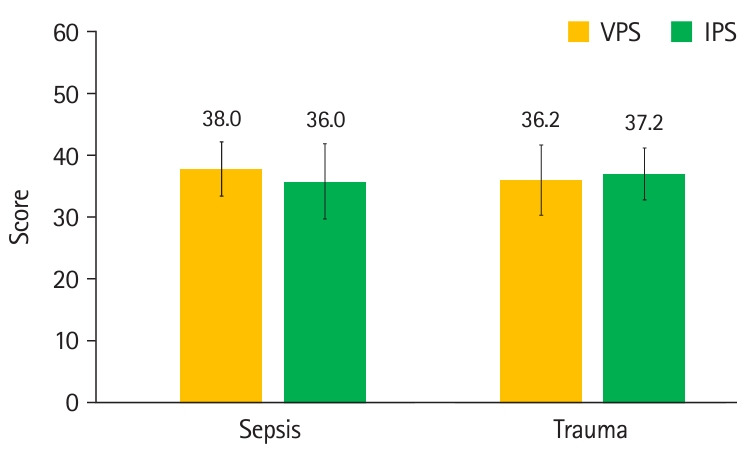
Comparison of overall mean checklist scores for sepsis and trauma scenarios between virtual patient simulation (VPS) and in-person simulation (IPS) groups. Whiskers show standard deviations.

The median SSLS scores were lower with VPS than with IPS (-3.0; 95% CI, -1.0 to -5.0) (Table 2, Fig. 3). The mean±SD sepsis checklist scores for VPS (38.0±4.4) and IPS (36.0±6.1) were not significantly different (2.0; 95% CI, -1.4 to 5.4), with a Cohen’s d of 0.38 (Table 3, Fig. 4). The mean±SD trauma checklist scores for VPS (36.2±6.0) and IPS (37.2±4.2) were not significantly different (-0.9; 95% CI, -4.2 to 2.3), with a Cohen’s d of 0.19.

**Fig. 5.**
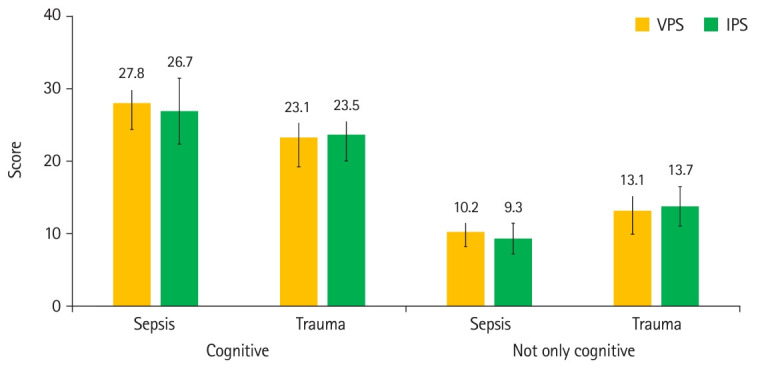
Comparison of mean checklist scores between virtual patient simulation (VPS) and in-person simulation (IPS) groups, stratified by learning taxonomy domains involved. Whiskers show standard deviations.

The mean checklist scores for items labeled as only cognitive were not significantly different for sepsis (1.1; 95% CI, -1.5 to 3.7) and trauma scenarios (-0.3; 95% CI, -2.8 to 2.1). Mean checklist scores for items labeled as not only cognitive were not significantly different for sepsis (0.9; 95% CI, -0.4 to 2.2) and trauma scenarios (-0.6; 95% CI, -2.4 to 1.3) (Fig. 5). The adjusted means were generated by analysis of covariance using imbalanced baseline characteristics as covariates. These were similar to the primary results, suggesting a minimal effect of imbalanced baseline characteristics (Table 4).

## Discussion

### Key results

The main objective of the study was to determine differences in learning outcomes between VPS and IPS for sepsis and trauma resuscitation. The main findings are that there was no significant difference in learning outcomes overall and when stratified by cognitive and not-only cognitive domains.

### Interpretation

The predicted benefit of a simpler simulation for novices did not clearly manifest in this study as a large improvement in overall sepsis and trauma resuscitation skills learning. Any true difference would be small, not observable with our sample size, and relatively insignificant from an educational standpoint. We investigated whether this lack of overall difference was due to the attenuation of cognitive benefits by poorer performance in non-cognitive domains, but found no domain-specific differences to support this.

The participants did not maximize the time given to them in VPS despite proctoring. The time-equivalent repetition enabled by VPS was modest, at 2 attempts per case, suggesting limited benefit through this mechanism. The short VPS time-on-task suggests both an explanation for lack of difference, and time efficiency in learning.

### Comparison with previous studies

Triangulated by reduced self-reported satisfaction, VPS appears to reduce the time-on-task learners are willing to spend. This is consistent with the surgical education literature, where learners prefer more sophisticated simulations with greater physical resemblance [13]. Such satisfaction is associated with improved knowledge and cognitive skill retention [14].

This lack of difference is consistent with Liaw et al. [3], who found no difference between VPS and IPS 2.5 months after the intervention, while VPS outperformed IPS 1–2 days after the intervention. This underscores the importance of differentiating retention and recall in education research.

### Limitations and strengths

There are multiple limitations of this study. First, it was a pilot study without a sample size calculation based on prior data on group performance, and it may have been underpowered. Even so, the detected differences were small (Cohen’s d=0.19 to 0.38), with no consistent direction of benefit within particular domains for VPS or IPS that would be hypothesis-generating. Assuming the observed differences hold steady, at an alpha of 0.05 and power of 80%, 632–1,114 participants would be needed to power a study for the trauma scenario. This likely exceeds the annual junior doctor census in most emergency departments, limiting feasibility.

Second, the confounders were unevenly distributed between groups despite randomization, which was likely attributable to the small sample size. Further studies could target a larger sample size to distribute confounders more effectively, or hold VPS and IPS sessions simultaneously or within the same day, though this was not feasible in our context. An adjusted analysis accounting for confounders demonstrated similar overall findings, with the intervention group not being a significant predictor of overall checklist scores.

The following are some strengths of this study. Validity evidence was gathered for the assessment tool, through a consensus process to sharpen content, scenario piloting, and tools to achieve standardization in scenario delivery and rater calibration, and good reliability as indicated by ICC. Retention was measured, rather than recall, with a median of over 40 days between intervention and assessment. A novel approach was taken by using learning outcome taxonomy domains to investigate mechanisms for difference, and not simply whether a difference existed. Allocation concealment and blinding were maintained, with no loss to follow-up.

### Generalizability

The generalizability of this study may be limited by a disproportionately large number of male participants undergoing a compulsory emergency department rotation before deployment to military service, which is mandatory for Singaporean men. Regarding gender effects, prior simulation literature suggests better initial performance in men that fades with training [13].

The findings are relevant to clinical educators globally, as sepsis and trauma are common and associated with significant and modifiable morbidity and mortality [15]. VPS is scalable solution, allowing its cost to be spread out among more learners across borders, and relevant to a wide range of educators addressing this challenge.

### Suggestions

Our findings suggest that VPS is a reasonable starting point for novices for sepsis and trauma resuscitation, producing similar learning outcomes to IPS, with the bonus of potential time savings. Further inquiry may explore whether the same applies to other tasks that integrate across domains, but with substantial cognitive contribution, and the sequential integration of VPS followed by IPS in a simulation curriculum.

Learner time-on-task does not automatically follow VPS implementation. Attention to design and implementation through a framework such as activity theory or motivational theories helps to understand drivers of VPS engagement and time-on-task that educators can influence, and represents an avenue for further research.

### Conclusion

Novices exhibited no large differences in overall and non-cognitive learning outcomes for sepsis and trauma resuscitation with VPS compared to IPS, though learners were less satisfied with VPS. VPS is a reasonable initial simulation training modality for novices learning sepsis with trauma resuscitation, but requires attention to fostering learner engagement and satisfaction.

## Figures and Tables

**Figure f6-jeehp-21-33:**
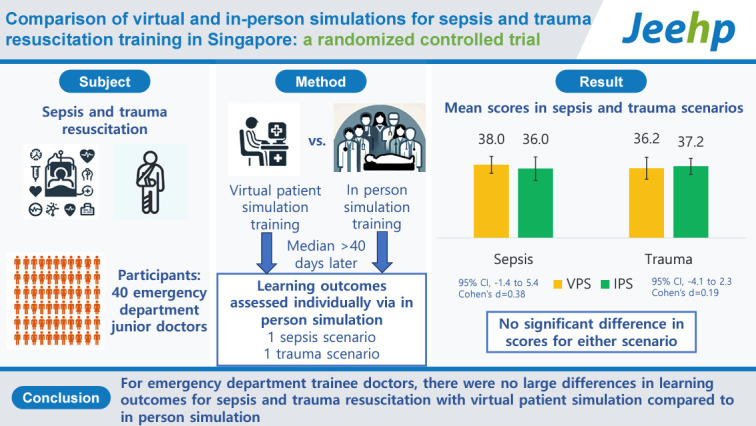


**Table 1.** Baseline characteristics

Characteristic	Virtual patient simulation	In-person simulation
Age (yr)	26.0 (25.0–29.0)	27.0 (26.0–28.0)
Sex		
Female	3 (15.8)	8 (38.1)
Male	16 (84.2)	13 (61.9)
No. of years post graduate	2.0 (2.0–3.0)	3.0 (2.0–4.0)
Months of prior experience in the emergency department as a doctor	0 (0.0–12.5)	3.0 (0.0–6.0)
No. of days between simulation training and assessment	41.0 (11.5–53.5)	49.5 (23.8–74.0)

Values are presented as median (interquartile range) or number (%) unless otherwise stated.

**Table 2.** Measurements from the Student Satisfaction with Learning Scale

Measurement	Median (interquartile range)		Difference
	Virtual patient simulation	In-person simulation	
Student Satisfaction with Learning Scale	20.0 (17.5–21.5)	23.0 (22.0–25.0)	-3.0 (95% CI, -1.0 to -5.0)

CI, confidence interval.

**Table 3.** Measurements from assessment scenario checklist items

Measurement	Mean±SD		Difference	Cohen’s d
	Virtual patient simulation	In-person simulation		
Sepsis scenario (overall, maximum 62 points)	38.0±4.4	36.0±6.1	2.0 (95% CI, -1.4 to 5.4)	0.38
Sepsis scenario (only cognitive, maximum 42 points)	27.8±3.6	26.7±4.5	1.1 (95% CI, -1.5 to 3.7)	
Sepsis scenario (not only cognitive, maximum 20 points)	10.2±2.0	9.3±2.1	0.9 (95% CI, -0.4 to 2.2)	
Trauma scenario (overall, maximum 62 points)	36.2±6.0	37.2±4.2	-0.9 (95% CI, -4.2 to 2.3)	0.19
Trauma scenario (only cognitive, maximum 40 points)	23.1±4.0	23.5±3.6	-0.3 (95% CI, -2.8 to 2.1)	
Trauma scenario (not only cognitive, maximum 22 points)	13.1±3.2	13.7±2.7	-0.6 (95% CI, -2.4 to 1.3)	

SD, standard deviation; CI, confidence interval.

**Table 4.** Adjusted analysis of checklist score means

Outcome	Unadjusted mean	Adjusted mean	Unadjusted mean difference	Adjusted mean difference
Sepsis checklist score			2.0 (95% CI, -1.4 to 5.4)	2.5 (95% CI, -1.0 to 6.0)
Virtual patient simulation	38.0	38.3		
In person simulation	36.0	35.8		
Trauma checklist score			-0.9 (95% CI, -4.2 to 2.3)	-1.4 (95% CI, -4.7 to 1.8)
Virtual patient simulation	36.2	36.0		
In person simulation	37.2	37.4		

CI, confidence interval.

## Footnotes

**Data availability**
Supplementary files are available from Harvard Dataverse: https://doi.org/10.7910/DVN/KHAYXN Dataset 1. The data file contains de-identified outcome assessment data of study participants.

## Supplementary materials

Supplementary files are available from Harvard Dataverse: https://doi.org/10.7910/DVN/KHAYXN

Supplement 1. Assessment scenario checklists with domains involved.

Supplement 2. Audio recording of the abstract.
