# Supplementary material for: Comparison of virtual and in-person simulations for sepsis and trauma resuscitation training in Singapore: a randomized controlled trial
Source: J Educ Eval Health Prof. 2024 Nov 18;21:33. doi: 10.3352/jeehp.2024.21.33 (PMC11647267; doi:10.3352/jeehp.2024.21.33)
Supplement: Supplementary file 2 — Supplement 1. Assessment scenario checklists with domains involved. [file jeehp-21-33-suppl1.docx]

**Supplement 1.** Assessment scenario checklists with domains involved

**Case 1: Severe community acquired pneumonia–simulated resuscitation checklist**

Domains involved are marked with 1, and domains not involved are marked with 0.

| No. | Action | Cognitive | Psychomotor | Affective |
| --- | --- | --- | --- | --- |
| 1 | Isolates patient in critical care isolation area | 1 | 0 | 0 |
| 2 | Ensures team and self-don personal protective equipment | 1 | 0 | 1 |
| 3 | Assesses airway and recognizes it is patent | 1 | 1 | 0 |
| 4 | Assesses breathing and recognizes respiratory distress with severe tachypnoea | 1 | 1 | 0 |
| 5 | Obtains vital signs including peripheral oxygen saturation | 1 | 0 | 0 |
| 6 | Provides oxygen via non-rebreather mask | 1 | 0 | 0 |
| 7 | Obtains history from patient:  - Breathlessness and its onset, chest pain  - Chills, cough, hemoptysis  - No orthopnea or lower limb swelling  - No contact history with others with COVID-19 or similar symptoms | 1 | 0 | 0 |
| 8 | Physical examination, including:  - Lung auscultation  - Cardiac auscultation  - Capillary refill time  - Jugular venous pulse  - Limb oedema  - Other sources of sepsis, e.g., abdominal examination for tenderness and guarding, skin and soft tissue | 1 | 1 | 0 |
| 9 | Obtains intravenous access | 1 | 1 | 0 |
| 10 | Initiates intravenous fluid resuscitation, eventually delivering 30 mL/kg of crystalloid | 1 | 0 | 0 |
| 11 | Obtains point of care testing:  - Electrocardiogram  - Arterial blood gas  - Lactate | 1 | 0 | 0 |
| 12 | Obtains appropriate laboratory testing:  - Full blood count  - Renal panel  - Troponin | 1 | 0 | 0 |
| 13 | Obtains chest X-ray | 1 | 0 | 0 |
| 14 | Correctly interprets electrocardiogram, recognizing sinus tachycardia and ST depressions | 1 | 0 | 0 |
| 15 | Correctly interprets arterial blood gas, recognizing:  - Hypoxemic and hypercapnic respiratory failure  - Metabolic acidosis | 1 | 0 | 0 |
| 16 | Correctly interprets chest X-ray, recognizing right sided air space opacities | 1 | 0 | 0 |
| 17 | Diagnoses severe community acquired pneumonia on basis of shock, respiratory failure, and organ dysfunction | 1 | 0 | 0 |
| 18 | Obtains blood cultures before antibiotics | 1 | 0 | 0 |
| 19 | Intravenous antibiotics for severe pneumonia based on local antibiogram | 1 | 0 | 0 |
| 20 | Recognizes need for intubation and ICU care for respiratory failure and sepsis | 1 | 0 | 0 |
| 21 | Applies pre-oxygenation and apneic oxygenation | 1 | 1 | 0 |
| 22 | Hemodynamic optimization before intubation. Checks for blood pressure response after fluids, and tries to optimize further. Options include:  - Intravenous fluids 1.5 to 2 L total  - Starts vasopressors via peripheral or central line | 1 | 0 | 0 |
| 23 | Assesses for anatomically difficult airway, and optimizes positioning, as determined by tragal line and sternum | 1 | 1 | 0 |
| 24 | Appropriate choice of induction and paralysis agents:  - Ketamine or etomidate for induction  - Succinylcholine or rocuronium for paralysis | 1 | 0 | 0 |
| 25 | Intubates manikin at first pass | 1 | 1 | 0 |
| 26 | Confirms placement with end-tidal CO_2_ detection | 1 | 0 | 0 |
| 27 | Gives post intubation sedation and analgesia | 1 | 0 | 0 |
| 28 | Attaches patient to and sets ventilator | 1 | 1 | 0 |
| 29 | Obtains post-intubation chest X-ray | 1 | 0 | 0 |
| 30 | Checks vital signs after intubation | 1 | 0 | 0 |
| 31 | Refers patient to ICU team for admission to ICU | 1 | 0 | 1 |

COVID-19, COVID-19, coronavirus disease 2019; ICU, intensive care unit.

**Case 2: Major trauma with pelvic fracture and splenic injury–simulated resuscitation checklist**

Domains involved are marked with 1, and domains not involved are marked with 0.

Tick the box that applies

| No. | Action | Cognitive | Psychomotor | Affective |
| --- | --- | --- | --- | --- |
| 1 | Assigns roles to team members | 1 | 0 | 1 |
| 2 | Activates general surgery trauma team to come to ED:  - At latest, general surgery trauma team should be activated once hypotension is picked up in ED | 1 | 0 | 0 |
| 3 | Ensures team and self-don personal protective equipment | 1 | 0 | 0 |
| 4 | Prepares equipment and resuscitation area:  - Bag valve mask, laryngoscope, endotracheal tube  - Equipment for intravenous access and fluids | 1 | 0 | 0 |
| 5 | Obtains handover from paramedic:  - Mechanism (and time) of injury  - Injuries  - Symptoms and signs  - Treatment | 1 | 0 | 0 |
| 6 | Assesses airway  - Recognizes it is patent based on patient speaking | 1 | 1 | 0 |
| 7 | Assesses breathing  - Inspection, palpation, auscultation  - Picks up left lower chest wall tenderness | 1 | 1 | 0 |
| 8 | Obtains vital signs | 1 | 0 | 0 |
| 9 | Assesses circulation:  - Looks for sources of bleeding: external, chest, abdomen, pelvis, long bones  - Recognizes likely pelvic injury | 1 | 1 | 0 |
| 10 | Obtains intravenous access with 2 large bore cannulas | 1 | 0 | 0 |
| 11 | Obtains blood and sends it for:  - Group and cross match  - Coagulation studies  - Renal panel  - Full blood count | 1 | 0 | 0 |
| 12 | Starts volume resuscitation with either blood products or maximum 500 mL of crystalloid | 1 | 0 | 0 |
| 13 | Activates massive transfusion protocol | 1 | 0 | 0 |
| 14 | Gives tranexamic acid | 1 | 0 | 0 |
| 15 | Obtains blood gas and lactate | 1 | 0 | 0 |
| 16 | Obtains extended FAST and recognizes intra-abdominal free fluid | 1 | 0 | 0 |
| 17 | Assesses disability:  - GCS  - Pupils  - Focal weakness | 1 | 1 | 0 |
| 18 | Obtains full exposure for examination | 1 | 1 | 0 |
| 19 | Carries out log roll with spinal precautions | 1 | 1 | 0 |
| 20 | Applies pelvic binder | 1 | 1 | 0 |
| 21 | Keeps patient warm with blankets | 1 | 0 | 0 |
| 22 | Gives opioid analgesia | 1 | 0 | 0 |
| 23 | Obtains chest X-ray and pelvis X-ray | 1 | 0 | 0 |
| 24 | Correctly interprets arterial blood gas, recognizing:  - Metabolic acidosis  - Hyperlactatemia | 1 | 0 | 0 |
| 25 | Correctly interprets chest X-ray as normal | 1 | 0 | 0 |
| 26 | Correctly interprets pelvis X-ray as open book fracture | 1 | 0 | 0 |
| 27 | Obtains an AMPLE history:  - Allergies  - Medications  - Past medical history  - Last meal  - Events surrounding injury | 1 | 0 | 0 |
| 28 | Brief secondary survey | 1 | 1 | 0 |
| 29 | Discusses case with trauma surgeon in person, accurately describing:  - Mechanism of injury  - Injuries identified  - Relevant investigation results (FAST, X-ray, blood gas)  - Treatments given | 1 | 0 | 1 |
| 30 | Refers patient to orthopedic surgery | 1 | 0 | 1 |
| 31 | Arranges for patient to be transferred to operating theatre for surgical hemostasis | 1 | 0 | 0 |

ED, emergency department; FAST, Focused Assessment with Sonography for Trauma; GCS, Glasgow Coma Scale.
